# Expression of the Tick-Associated Vtp Protein of *Borrelia hermsii* in a Murine Model of Relapsing Fever

**DOI:** 10.1371/journal.pone.0149889

**Published:** 2016-02-26

**Authors:** Renee A. Marcsisin, Eric R. G. Lewis, Alan G. Barbour

**Affiliations:** Department of Microbiology and Molecular Genetics and Department of Medicine, University of California Irvine, Irvine, California, United States of America; University of Toledo College of Medicine and Life Sciences, UNITED STATES

## Abstract

*Borrelia hermsii*, a spirochete and cause of relapsing fever, is notable for its immune evasion by multiphasic antigenic variation within its vertebrate host. This is based on a diverse repertoire of surface antigen genes, only one of which is expressed at a time. Another major surface protein, the Variable Tick Protein (Vtp), is expressed in the tick vector and is invariable at its genetic locus. Given the limited immune systems of ticks, the finding of considerable diversity among the Vtp proteins of different strains of *B*. *hermsii* was unexpected. We investigated one explanation for this diversity of Vtp proteins, namely expression of the protein in mammals and a consequent elicitation of a specific immune response. Mice were infected with *B*. *hermsii* of either the HS1 or CC1 strain, which have antigenically distinctive Vtp proteins but otherwise have similar repertoires of the variable surface antigens. Subsequently collected sera were examined for antibody reactivities against Vtp and other antigens using Western blot analysis, dot blot, and protein microarray. Week-6 sera of infected mice contained antibodies that were largely specific for the Vtp of the infecting strain and were not attributable to antibody cross-reactivities. The antibody responses of the mice infected with different strains were otherwise similar. Further evidence of *in vivo* expression of the *vtp* gene was from enumeration of cDNA sequence reads that mapped to a set of selected *B*. *hermsii* genes. This measure of transcription of the infecting strain’s *vtp* gene was ~10% of that for the abundantly-expressed, serotype-defining variable antigen gene but similar to that of genes known for *in vivo* expression. The findings of Vtp expression in a vertebrate host and elicitation of a specific anti-Vtp antibody response support the view that balancing selection by host adaptive immunity accounts in part for the observed diversity of Vtp proteins.

## Introduction

Arthropod-borne, pathogenic spirochetes of the genus *Borrelia* adjust expression of their genes for the particular host of the moment [1]. For example, *Borrelia burgdorferi*, a cause of Lyme disease, abundantly expresses the OspA surface protein in the unfed tick, but down-regulates expression with the entry of blood into the vector’s intestine, and this reduction continues after transmission into a vertebrate host (reviewed in [2]). As OspA expression declines, expression of another surface protein, OspC, rises and persists for a few weeks. As befits a protein that is primarily expressed in an invertebrate with a limited immune system [3], there are few differences between the OspA proteins of different strains of the species [4, 5]. Antibodies against OspA are seldom found in naturally-infected reservoirs, such as the rodent *Peromyscus leucopus* [6]. The limited inter-strain diversity of OspA contrasts with the marked diversity of OspC proteins [5, 7], which commonly elicit antibodies in natural vertebrate hosts [8] and appear to be under selection by adaptive immunity [9].

A second major clade of *Borrelia* species include the agents of relapsing fever, such as *B*. *hermsii* and *B*. *turicatae* in North America and *B*. *duttonii* and *B*. *crocidurae* in Africa [10]. Another species in this clade is *B*. *miyamotoi*, a recently recognized human pathogen [11]. Among relapsing fever group species with sequenced genomes, none demonstrably have coding sequences that are homologous to OspA [12, 13]. But relapsing fever agents do have the genetic capacity to sequentially produce a variety of Vsp and Vlp proteins [14], which are homologous to OspC and variable lipoprotein surface-exposed (VlsE) proteins of *B*. *burgdorferi*, respectively [15, 16]. Vsp and Vlp proteins together are called “variable membrane proteins” (VMP) and are lipoproteins that are encoded by multiple paralogous genes on linear plasmids [17].

There is only one type of *ospC* gene in cells of a *B*. *burgdorferi* strain [18]. Sequences of *ospC* genes [19, 20], as well as profiles of antibody reactivity to OspC proteins [21, 22], serve to distinguish *between strains* of Lyme disease agents. In contrast, there are several types of *vsp* and *vlp* genes in any given strain of a relapsing fever species, and expression of one or the other serves to distinguish between serotypes *within a strain* [23]. In each genome the several different *vsp* and *vlp* alleles are denoted by an appended number, as in “*vsp6*”. Through a duplicative transposition event that replaces the sequence downstream of a single promoter, expression of a single new allele is achieved [24]. The promoter for the *vsp* or *vlp* gene at this locus is active when the spirochetes are in a vertebrate but it is effectively silent when they are in the tick [25, 26].

Another member of the Vsp/OspC protein family is singularly designated the Variable Tick Protein, or Vtp [27]. As is the case for *B*. *burgdorferi*’s *ospC* gene, there is only one copy of the *vtp* gene in *B*. *hermsii*’s genome [15, 23, 27]. This gene and its promoter are located on a different linear plasmid than the one that bears the *vsp/vlp* promoter [28]. Vtp proteins are further distinguished from Vsp proteins by a different signal peptide for the lipoprotein [15].

*B*. *hermsii* cells in an unfed tick’s salivary glands express Vtp and little or no Vsp or Vlp [1]. Vtp is not required for migration to and colonization of the salivary glands, but it is necessary for transmission of *B*. *hermsii* from the tick to a mouse [26]. Presumably, cells bearing Vtp proteins enter the vertebrate as soon as saliva flows at the bite. But Vtp^+^VMP^-^ cells delivered by needle injection or tick bite were short-lived in the blood and did not achieve as high densities as Vtp^-^VMP^+^ cells in mice [26, 28, 29]. In experimental infections initiated by tick bite, Vtp^+^ cells were undetectable in blood smears that were otherwise rich in other spirochetes expressing either a Vsp or Vlp [25, 26].

The association of Vtp expression with the unfed tick environment calls to mind the conditions for expression of OspA by *B*. *burgdorferi* [30]. But in contrast to OspA proteins, which are nearly identical in sequence between different strains [5], the Vtp proteins of different strains of *B*. *hermsii* are as diverse as strain-defining OspC proteins of *B*. *burgdorferi* [27]. Could Vtp be expressed by the spirochetes for sufficiently long after onset of infection to elicit an immune response in vertebrate hosts? Raffel et al. reported the presence of antibodies to Vtp in mice inoculated with cells constitutively expressing Vtp and, in lower amounts, in mice infected with wild type organisms [26]. But specificity of the antibodies binding to Vtp was not reported in that study. Conceivably, the observed binding of antibodies to Vtp was instead the consequence of cross-reactivity with one or more homologous Vsp proteins expressed during infection. Here, we report of an investigation, which was prompted by a serendipitous observation, that addressed this question. We provide additional evidence that Vtp is expressed in mammals as well as in ticks and that specific anti-Vtp antibodies are elicited during infection of the mouse. These findings have implications for understanding the population structures of *B*. *hermsii* and other relapsing fever agents.

## Materials and Methods

### Strains and culture conditions

The bacteria included serotypes 7 and 19 *B*. *hermsii* strain HS1 from Browne Mountain, Spokane County, Washington [31, 32], serotype 1 *B*. *hermsii* strain CC1 from Mono County, California [33], and *B*. *burgdorferi* strain B31 (ATCC 35210). The Browne Mountain isolate of strain HS1 has the same GenBank taxid number (314723) as this strain’s DAH isolate [13]. The HS1 serotype 33 cell line that produced Vtp constitutively during in vitro cultivation has been described [28], and this provided the wild-type Vtp^+^ population. A spontaneous VMP null mutant of serotype 33 was described by Marcsisin et al. [33]. The mutant has a frame shift (FS) at position 83 of the coding sequence, leading to a stop codon beginning at position 90 (accession number JN232112), and a processed lipopeptide of only 12 amino acids. Here it is designated “HS1 *vtp*FS83” and had been cloned twice in succession by limiting dilution before the present study. A Vtp^-^ cell line with a kanamycin cassette knock-out of the *vtp* gene of strain HS1 was described by Lewis et al. [34] and is here designated “HS1 *vtp*::kan”. Spirochetes were grown in BSK II medium with 6% rabbit serum [35]. Cells were counted with a Petroff-Hausser chamber under phase-contrast microscopy. Spirochetes in broth medium were harvested by centrifugation at 9,500 x *g* for 20 min.

### Mouse infections

All animal work was conducted with specific approval of University of California Irvine’s Institutional Animal Care and Use Committee (protocol 2080–1999), which specified early euthanasia for animals who became severely ill or moribund during the course of the experiment, as described below. Mice were housed under ABSL2 containment in an Association for Assessment and Accreditation of Laboratory Animal Care-approved facility. This study was carried out in accordance with the recommendations in the Guide for Care and Use of Laboratory Animals of the National Institutes of Health. All *Mus musculus* were purchased from Charles River Laboratories (Wilmington, MA). To provide in vivo-propagated cells for the infections, 6-week-old CB17 Severe Combined Immunodeficiency (SCID) mice were inoculated intraperitoneally with 1–5 *B*. *hermsii* cells of a known serotype from frozen stocks. Inoculated mice were examined daily for hypoactivity, reduced responsiveness, and/or inability to drink fluids or eat. When there were ~10^8^ spirochetes per ml of blood, or before that if there were signs of severe illness, the mice were euthanized by carbon dioxide inhalation overdose followed by exsanguination and cervical dislocation. Citrated blood was centrifuged at 100 x *g* for 3 min. For studies of the antibody response, the plasma supernatant was diluted with BSK II medium to provide individual inocula of 10–50 spirochetes in 50 μl volumes for intraperitoneal injection of female 8-week-old BALB/c mice. Blood was collected on day 42 by terminal cardiac puncture. The plasma supernatant fraction obtained by centrifugation at 13,000 x *g* for 5 min was stored at -80°C. For the RNAseq experiment SCID mice were infected as described and citrated whole blood was collected when the spirochete density reached 10^8^ cells per ml. In these experiments none of the mice died from the infection, became moribund, or showed signs of severe illness.

### Antiserum and monoclonal antibodies

The origin of monoclonal antibody H4825 to Vtp protein and its production in mouse ascitic fluid was previously described [35]. This antibody is specific for Vtp proteins categorized as type 6, which includes HS1 [27]. The monoclonal antibody to Alp of *B*. *hermsii* was previously described [33]. For polyclonal antisera female 6- to 8-week-old BALB/c were inoculated intraperitoneally with 10^8^ viable, culture-grown *B*. *hermsii* cells in 100 μl phosphate-buffered saline on day 0. On day 30, the mice were boosted by intravenous injection with the same dose, and blood was collected 12 d after the boost.

### Polyacrylamide gel electrophoresis (PAGE) and Western blot (WB)

PAGE and WB analyses were carried out as described [36]. Mouse sera and ascitic fluids with monoclonal antibodies were used at dilutions of 1:100 and 1:500, respectively. Bound antibodies were detected with alkaline phosphatase-conjugated goat anti-mouse IgG (Kirkegaard and Perry, Gaithersburg, MD) at a 1:10,000 dilution, and the 1-Step NBT/BCIP substrate solution (Pierce) was used for colorimetric detection. Blots were digitally scanned, and the pixels in bands on the blots were measured by the gel analysis protocol in the ImageJ suite version 1.48 (http://rsb.info.nih.gov/ij). For PAGE and WB analysis of detergent fractions, cell pellets in microcentrifuge tubes were resuspended at a final concentration of 5 x 10^8^ cells per ml of ice-cold PBS with 2% Triton X-114 (Sigma-Aldrich, St. Louis, MO). The suspension was then subjected to the fractionation procedure of Nally et al. [37] with protein precipitation in acetone.

### PCR and DNA cloning

Genomic DNA was extracted from culture-grown *B*. *hermsii* using a Qiagen (Valencia, CA) DNeasy Blood and Tissue kit as described [34]. Selected fragments were amplified by PCR with Phusion High-Fidelity DNA Polymerase (New England BioLabs, Ipswich, MA). PCR of the *vtp* genes was carried out as described [33, 34]. Full-length coding sequences for other genes were similarly amplified using gene-specific primers as described [38]. For lipoproteins the signal peptide was excluded and the N-terminal cysteine of the processed lipoprotein was replaced by a methionine and alanine. The PCR products were mixed with a N-terminal His tagged pXT7 linear vector and transformed into competent cells of *E*. *coli* strain DH5α [39]. Plasmid DNA was isolated using the High Pure Plasmid Isolation kit (Roche Applied Science, Indianapolis, IN) and sequenced by the Sanger method at GENEWIZ (San Diego, CA).

### Recombinant proteins and microarray printing

Plasmids were transformed into *E*. *coli* BL21(DE3)*pLysS* cells (EMD Chemicals, Gibbstown, NJ). Transformants were cultivated in MagicMedia *E*. *coli* Expression Medium (Invitrogen, Carlsbad, CA). The cell culture was centrifuged at 9,500 x g for 5 min. The pellet was resuspended in FastBreak Cell Lysis Reagent (Promega, Madison, WI) with 25 U per ml of Benzonase Nuclease & EDTA-free Protease Inhibitor Cocktail (EMD Chemicals), and then lysed by gentle shaking for 25 min at 22°C. The lysate was centrifuged at 9,500 x *g* for 5 min. The protein with its His-tag was purified with the MagneHis Protein Purification System (Promega, Madison, WI) and then concentrated with Amicon Ultra-4 Centrifugal Filter Unit (Millipore, Billerica, MA) in storage buffer as described [38, 40]. Sample purity of at least 90% was confirmed by PAGE, and protein concentrations were determined by BCA Protein Assay kit (Pierce, Rockford, IL). Purified recombinant proteins at concentrations of 20 μg/ml, except as noted, were printed in quadruplicate on nitrocellulose-coated glass FAST slides (Whatman, Piscataway, NJ) in 1.5-nanoliter volumes using an Omnigrid 100 apparatus (Digilab, Holliston, MA). An anti-histidine tag monoclonal antibody (Sigma, St. Louis, MO) at a 1:1000 dilution confirmed protein presence on the array. Negative controls were spots of storage buffer, PBS, and *B*. *burgdorferi* proteins BBK07 and BBK19, which do not have homologs in relapsing fever *Borrelia* species [41].

### Dot blot

Purified recombinant protein at 20 μg/ml was spotted in 1 μl volumes onto Whatman Optitran BA-S 85 0.45 μm nitrocellulose membranes. Membranes were blocked with 5% (wt/vol) non-fat dry milk in 1 mM Tris, pH 7.6–150 mM NaCl-0.05% Tween 20 (TST) for 1 h, and then incubated with TST with a 1:100 dilution of serum for 1 h at 22°C. Bound antibodies were detected with the alkaline phosphatase-conjugated goat anti-mouse IgG at a 1:10,000 dilution in TST and then development in a 1-Step NBT/BCIP substrate solution (Pierce).

### Microarray analysis

The procedure essentially followed the method of Baum et al. [40]. In brief, the slides were blocked with 0.3% non-fat dry milk in TST for 1 h. Sera were diluted 1:100 in 0.3% non-fat dry milk in TST and incubated with an *E*. *coli* lysate suspension (MCLAB, San Francisco, CA) at 1:10 dilution for 30 min at 22°C. The blocked slides were then incubated with the post-absorption sera solutions for 10 h at 4°C and subsequently washed seven times in TST. Bound antibodies were detected with Cy5-conjugated goat anti-mouse IgG (Jackson ImmunoResearch, West Grove, PA) at a 1:200 dilution in TST in the dark for 1 h at 22°C on a rocking platform. After the slides were washed with TST, they were scanned in a ScanArray 4000 laser confocal scanner (GSI Lumonics, Billerica, MA). Fluorescence intensities were measured by using Quantarray software (GSI Lumonics).

### Sequences

The complete chromosome sequence of *B*. *hermsii* strain CC1 has GenBank accession number CP011060. The sequences of the Vtp proteins of strains HS1 and CC1 have accession numbers JN232111 and JF737018, respectively. The accession numbers of other genes and selected other loci are given in Table 1. The 2.6 Gb *Mus musculus* sequence of Genome Reference Consortium Mouse Build 38 (GCA_000001635.2) was used as reference.

**Table 1:** RNA-Seq with selected genes of *B*. *hermsii* CC1 in blood of mice.

Rank	Gene (strain/species)	Length (bp)	Accession no.^a^	Log_10_ reads/1000 bp
1	*flaB*	1002	JF737019	3.44
2	*vsp1*	443	EF154507	3.19
3	*groEL*	1665	CP011060	2.46
4	*vtp* (CC1)	449	JF737018	2.40
5	*fbpC*	1125	KF537350	2.35
6	*glpQ*	1020	EF601049	2.27
7	*fhbA*	486	EF195129	2.18
8	*rpsB*	765	CP011060	2.14
9	*rplB*	846	CP011060	2.14
10	*p66*	1778	EF160110	2.11
11	*rpsD*	627	CP011060	2.09
12	*rplC*	630	CP011060	2.06
13	*rpsC*	831	CP011060	2.03
14	*recA*	1071	CP011060	2.00
15	*purB*	1395	EF191164	1.98
16	*ftsA*	1242	CP011060	1.98
17	*rplA*	675	CP011060	1.93
18	*purA*	1284	EF191164	1.90
19	*thyX*	771	EF153513	1.86
20	*vsp8*	455	EF185890	1.86
21	*vsp13*	446	EF153509	1.80
22	*vsp11*	437	EF185889	1.79
23	*ftsY*	855	CP011060	1.68
24	*vsp58*	443	EF156411	1.63
25	*vsp22*	455	EF156411	1.62
26	*vsp26*	449	EF153487	1.55
27	*vsp2*	446	EF153508	1.30
28	*vsp3*	446	EF158032	0.35
29–35^b^	*vtp* (HS1)	446	JN232111	ND^c^
29–35	*vtp* (LPO)	443	EF565825	ND
29–35	*vtp* (Owl)	446	GQ175066	ND
29–35	*vsp6* (HS1)	446	L33898	ND
29–35	*vspA* (Bt)	443	U85413	ND
29–35	*vsp1* (Bm)	446	KF031441	ND
29–35	*ospC* (Bb)	428	AE00079	ND

^a^GenBank accession number

^b^Assigned same range of ranks

^c^ND, none detected

### RNA-Seq

Total RNA was isolated from ~1 ml of citrated whole blood from each mouse using a RNeasy Mini Kit (Qiagen, Valencia, CA, USA) with modification of the protocol for automated use in a Qiacube instrument (Qiagen). After RNA was eluted from the columns in 50 μl of RNase-free water, residual DNA was removed by treatment with DNase I during further processing with a RNA Clean & Concentrator-5 kit (Zymo Research). The sample was depleted of ribosomal RNA by incubation of the solution at 50°C with the beads of Ribo-Zero Magnetic Kit for Gram-positive bacteria (Epicentre, Madison, WI) followed by ethanol precipitation. Synthesis of cDNA with random hexamer primers was carried out with a Maxima First Strand cDNA Synthesis Kit (Thermo Fischer Scientific). The resultant cDNA was sheared enzymatically, and adapters were ligated to the ends with an Ion Express Plus Fragment Library Kit (Life Technologies, Carlsbad, CA), and products were size selected with the E-Gel electrophoresis system and a 2% Size-Select gel (Life Technologies). The library was attached to Ion Sphere Particles using an Ion Torrent OneTouch 200 Template kit (Life Technologies), and an Ion Torrent OneTouch device performed emulsion PCR on the sample. The resultant product was then enriched using an Ion Torrent OneTouch ES apparatus and then sequenced on an Ion Torrent Personal Genome Machine with Ion 316 chips and Ion PGM 200 Sequencing Kit (Life Technologies). After filtering reads of *M*. *musculus* sequences, the RNA-Seq program in the CLC Genomics Workbench, version 8.1 (Qiagen) was used to enumerate reads mapping to reference sequences with these criteria: minimum length fraction of 1.0, minimum similarity fraction of 0.9, and costs of 2, 3, and 3 (out of 3) for mismatches, insertions, and deletions, respectively.

### Statistics

Parametric (*t* test) and nonparametric (Kruskal-Wallis rank test) analyses of continuous data were carried out with Stata/MP version 10.1 (StataCorp, College Station, TX) or Systat version 13 (Systat Software, Chicago, IL). Significance tests were two-sided. For ratios, the antilogs of the calculated means and 95% confidence intervals (CI) of log-transformed data were used and yielded asymmetric CI.

## Results

### Antibodies to Vtp in mice inoculated with HS1 *vtp*FS83 cells

The study began as a follow-up of an unexpected finding. As reported by Marcsisin et al. [33], mice were immunized with viable cells of the VMP-less *vtp*FS83 mutant of strain HS1, and the post-boost sera were used in microagglutination and western blot assays. This experiment led to identification and characterization of the arthropod-associated lipoprotein Alp on the surface of the VMP-less cells [33]. But we also found that antisera to *vtp*FS83 cells agglutinated wildtype Vtp^+^ cells by a means not attributable to anti-Alp antibodies [42]. One explanation was that immunization with these cells elicited anti-Vtp antibodies.

**Fig 1:**
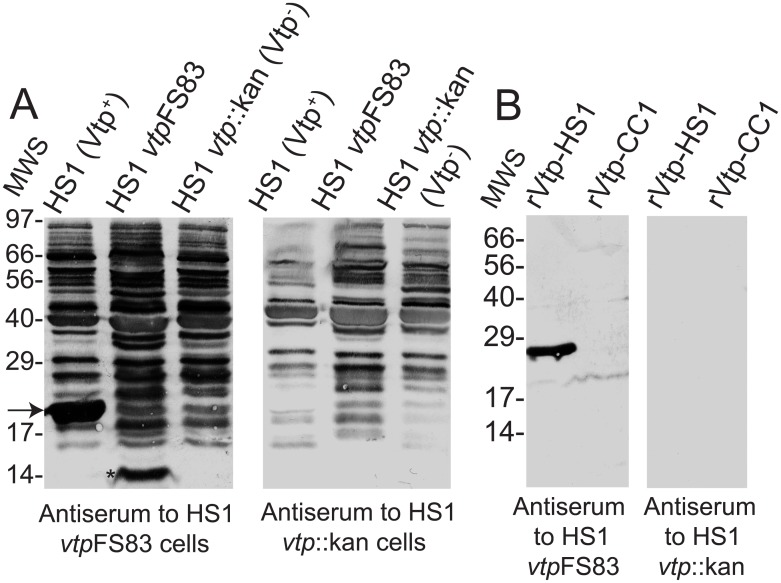
Western blot (WB) analyses of antisera to whole cells of *B*. *hermsii*. Panel A. Western blot analysis of whole cell lysates of in vitro cultures of strain HS1 *B*. *hermsii* isolates Vtp^+^ wildtype, *vtp*FS83 mutant, and *vtp*::kan knock-out. Blots were incubated with polyclonal mouse antisera to either the mutant or knock-out. Bound antibodies were detected with alkaline phosphatase-labeled anti-mouse immunoglobulin. An immunoreactive band in the WT lysate that was of the same size as Vtp is indicated by an arrow. A band corresponding to the Alp protein in the mutant lysate is indicated an asterisk. Molecular weight standards (MWS) in kilodaltons are shown on the left. Panel B. By Western blot analysis antibodies in the antiserum to *vtp*FS83 cells bound to purified recombinant Vtp (rVtp) of strain HS1 but not rVtp of strain CC1. Binding of antibodies in the antiserum to *vtp*::kan knock-out cells to either Vtp was not detected. Molecular weight standards (MWS) in kilodaltons are shown on the left.

This conjecture was investigated first by WB analysis of pooled sera against whole cell lysates of 3 isolates of strain HS1: wildtype, *vtp*FS83 mutant, or *vtp*::kan knockout. We compared the reactivities of antiserum to *vtp*FS83 cells with those of antiserum to *vtp*::kan cells (panel A of Fig 1). Both sets of antisera contained antibodies to numerous cellular proteins. As expected, the anti-mutant sera had antibodies to a component with an apparent molecular weight of ~14 kDa, which was identified with a monoclonal antibody as the Alp protein [33]. Notably the antiserum to *vtp*FS83 cells, but not antiserum to *vtp*::kan knock-out cells, contained antibodies to a protein of the expected size of Vtp in the Vtp^+^ wildtype cells.

The presence of antibodies to Vtp in the antiserum to *vtp*FS83 cells was confirmed by the binding of antibodies in that serum to recombinant Vtp of strain HS1 (panel B of Fig 1). In contrast, the antiserum raised in response to injection of the *vtp*::kan knock-out cells showed no detectable binding of Vtp in the blots. The specificity of these anti-Vtp antibodies for the Vtp of injected strain, HS1, was indicated by the absence of detectable binding to Vtp of strain CC1. The Vtp proteins of HS1 and CC1 strains differ at 68 (35%) of 197 amino acid positions beyond the N-terminal cysteine of the processed lipoprotein.

### *In vitro* expression of Vtp by HS1 *vtp*FS83 cells

**Fig 2:**
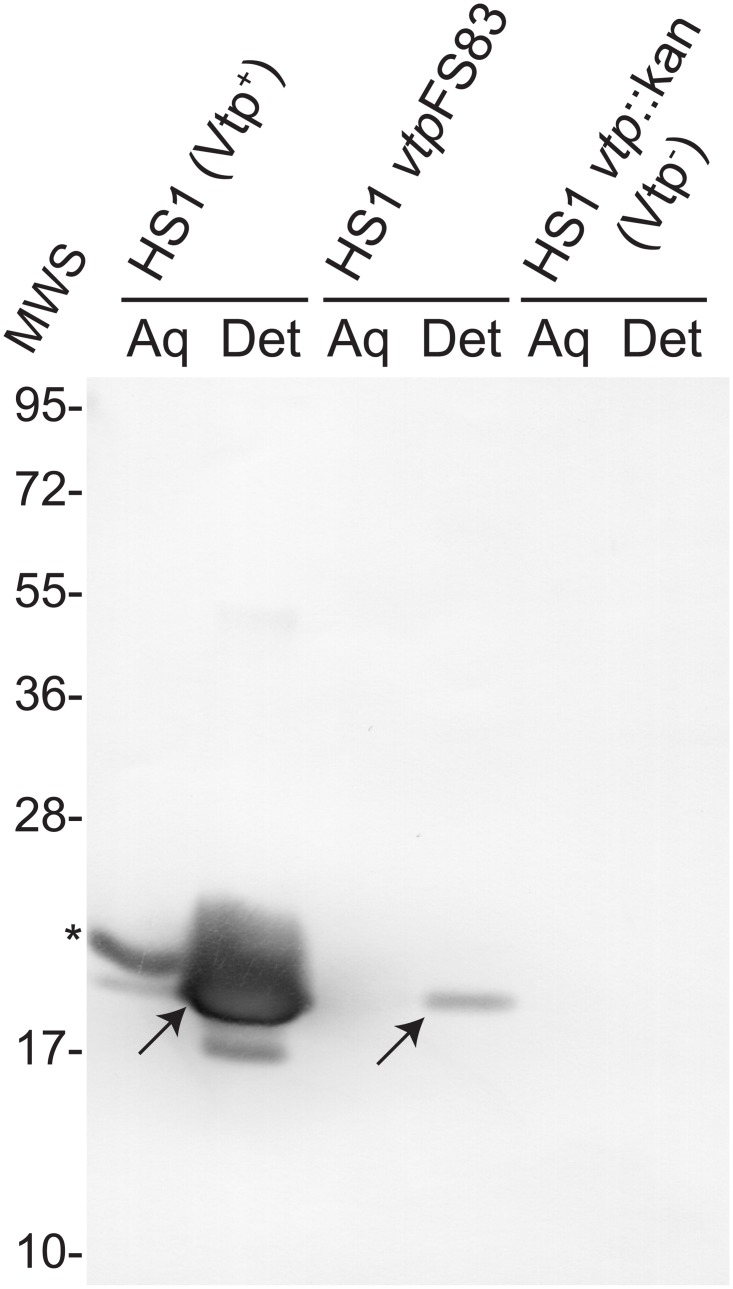
Western blot analysis with anti-Vtp monoclonal antibody of aqueous (Aq) or detergent (Det) fractions of Triton X-114 extractions of cells *B*. *hermsii* HS1 wildtype, *vtp*FS83 mutant, and *vtp*::kan knock-out. Bands that correspond to the predicted migrations of unlipidated preprotein and lipidated processed Vtp are indicated by an asterisk and arrows, respectively. Molecular weight standards (MWS) in kilodaltons are shown on the left.

With this additional evidence that *vtp*FS83 cells produced Vtp in some unknown amount, we enriched for membrane-associated proteins by a Triton X-114 extraction of in vitro-cultivated cells of the *vtp*FS83 mutant and *vtp*::kan knock-out. Equivalent amounts of proteins of aqueous and detergent fractions were subjected to WB analysis with a monoclonal antibody to the Vtp protein of HS1 (Fig 2). As expected, an immunoreactive band of the predicted size of Vtp was abundantly present the detergent fraction of wildtype cells. The slower migrating immunoreactive band in the aqueous fraction of the wildtype cells was consistent in size with the unlipidated Vtp preprotein [15]. An immunoreactive band was also seen in the detergent fraction of the *vtp*FS83 mutant cells but not in either detergent or aqueous fraction of the *vtp*::kan cells.

### Strain-specific antibodies to Vtp in infected mice

The demonstration of in vitro expression of Vtp by the mutant, albeit at low levels, meant that in vivo expression of the *vtp* gene by mutant cells was plausible, and that this circumstance could account for the anti-Vtp antibodies observed in the sera of inoculated mice (Fig 1). Alternatively, the observed binding of antibodies to Vtp could have been the consequence of cross-reactivity of antibodies elicited by one or more of Vsp proteins, with which Vtp is homologous [15].

**Fig 3:**
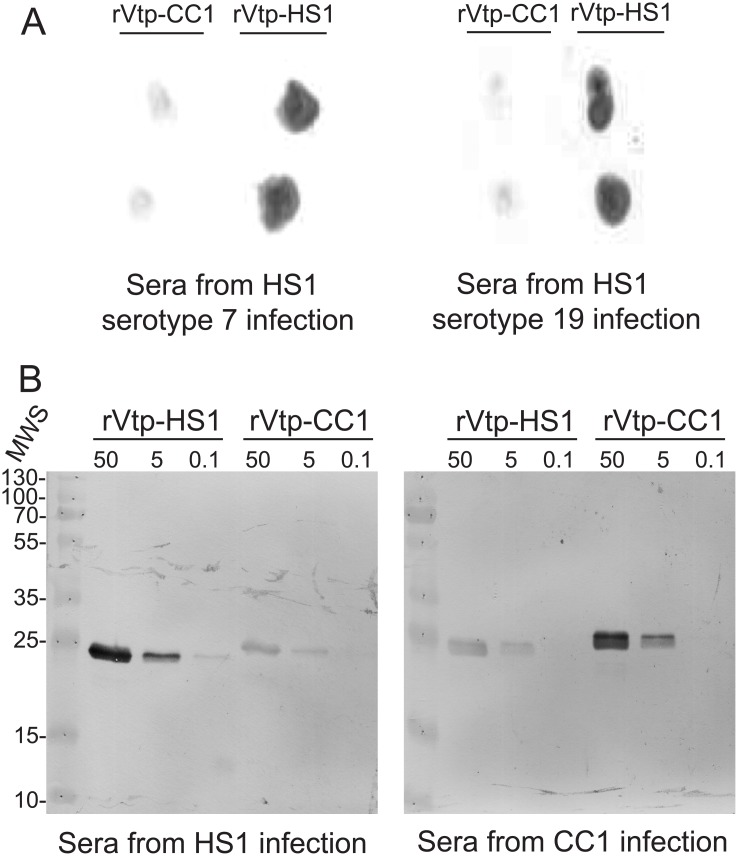
Antibodies to Vtp in sera from infected mice with different serotypes of *B*. *hermsii*. Panel A. Dot blot analysis of binding of antibodies in pooled sera from mice infected for 6 weeks with either serotype 7 or serotype 19 to recombinant Vtp proteins from either strain HS1 or CC1 of *B*. *hermsii*. Purified proteins at a concentration of 20 μg/ml were spotted onto nitrocellulose membranes in duplicate and then incubated with sera. Panel B. Western blot analysis of pooled week-6 sera from infected mice to recombinant Vtp proteins at concentrations of 50, 5, or 0.1 μg/ml. Molecular weight standards in kilodaltons are shown on the left.

We first determined whether infections initiated with low doses of *B*. *hermsii* of different serotypes elicit antibodies against Vtp. Two groups comprising 5 mice each were infected with ~50 cells of either serotype 7 or serotype 19 of strain HS1 and then bled after 6 weeks. Infection was confirmed by examination of tail vein blood by phase microscopy. The sera from the two different infections were pooled and then used in dot blot assays. Antibodies in post-infection sera qualitatively bound in greater amounts to Vtp of strain HS1 than to Vtp of strain CC1 (Panel A of Fig 3).

The specificity of the Vtp reactive antibodies elicited during infection was further assessed with addition of week-6 sera obtained from 5 mice similarly infected with ~50 cells of strain CC1. Recombinant Vtp proteins of HS1 and CC1 Vtp at concentrations of 50, 5, and 0.1 μg/ml were subjected to SDS-PAGE and Western blot analysis with pooled week-6 sera at 1: 100 dilution from the 5 mice infected with serotype 19 of strain HS1 or strain CC1 infection (Panel B of Fig 3). For the HS1 sera, the pixels per band for the 50, 5, and 0.1 concentrations of HS1 Vtp were 7762, 2630, and 155, respectively, and the corresponding values for CC1 Vtp were 912, 288, and 0. For the CC1 sera the values for HS1 Vtp were 2818, 708, and 0, and the values for CC1 Vtp were 6998, 2630, and 0. Antibodies in sera from uninfected mice showed no binding in the blots (data not shown). This experiment was further evidence of an immune response to Vtp during infection with other serotypes and of the specificity of the antibodies for the Vtp of the infecting strain.

### Antibody binding to a protein microarray

The comparative binding of antibodies in week-6 sera from 4 mice infected with serotype 7, 4 mice infected with serotype 19, and 5 mice infected with CC1, together with 3 sera from uninfected mice, was next examined with a protein microarray with individual mouse sera. The recombinant protein antigens were the following: FlaB flagellin proteins of *B*. *hermsii* and *B*. *burgdorferi*; the Vtp proteins of strains HS1 and CC1; Vsp2, Vsp11, Vsp13, Vlp4, Vlp9, Vlp12, and Vlp18 of *B*. *hermsii*; and OspC type A of *B*. *burgdorferi*. While the Vtp proteins of strains HS1 and CC1 are substantially different in sequence (S1 Fig), the FlaB proteins and the orthologous Vsp and Vlp proteins of these two strains are identical or near-identical. By *t*-test the antibody binding values for the sera from mice infected with serotypes 7 or 19 did not significantly differ at the 0.05 level for any of the 12 antigens. Accordingly, the results for the combined group of 8 HS1-infected mice were used for the comparison with the 5 sera from strain CC1 infection.

By the null hypothesis of no difference in antibody reactivities against two Vtp proteins for the different infecting strains, the average of the ratios of the binding values, i.e. Vtp_HS1_/Vtp_CC1_, would expected to be ~1. As internal controls, we used ratios of bindings for 3 other antigen pairs. The first control was the ratio of binding to the flagellin FlaB of *B*. *hermsii* to that for the FlaB protein of *B*. *burgdorferi*. But the proteins differ at 9% of residues, so any binding of cross-reactive antibodies to the *B*. *burgdorferi* FlaB was expected to be less than to the array’s *B*. *hermsii* FlaB. Since the FlaB proteins of strains HS1 and CC1 differed at only 1 of 334 amino acids, the FlaB proteins for each strain should be near equivalent in their reactivities with the sera from the different infections. Two other control antigen pairs were identical or near-identical between strains HS1 and CC1: the Vsp11 and Vsp13 proteins, which differ at 43% of residues, and the Vlp9 and Vlp12 proteins, which differ at 21% residues. These distances bracketed the sequence difference of 35% between the Vtp proteins of strains HS1 and CC1.

Table 2 summarizes the results for the four antigen pairs for the mice infected with HS1 or CC1. There was minimal or no binding of the antibodies of the uninfected animals to these proteins on the array (data not shown). There was essentially no difference in the sets of sera in their relative reactivities to the FlaB proteins or to the Vsp proteins. There was greater reactivity against the Vlp9 protein than to the Vlp12 protein by a 3-to-4-fold margin, but this ratio was not significantly different between infecting strain. Only for the pair of Vtp proteins did the two sets of sera reciprocally differ significantly in strain-specific ways by both parametric and non-parametric tests. These array results confirmed the evidence from the dot blot and Western blot analyses on a Vtp-specific immune response in infected mice.

**Table 2:** Protein array reactivities of sera from mice infected with HS1 or CC1 of *B*. *hermsii*.

Antigen pairs	Ratio (95% CI)^a^ HS1 mice (n = 8)	Ratio (95% CI) CC1 mice (n = 5)	*p* value *t* test	*p* value K-W test^b^
FlaB (Bh)/FlaB (Bb)^c^	3.1 (1.5–6.2)	2.6 (1.3–5.3)	0.80	1.0
Vtp (HS1)/Vtp (CC1)	15.9 (4.7–54.0)	0.41 (0.07–2.6)	0.005	0.013
Vsp11/Vsp13	1.0 (0.3–3.0)	0.9 (0.2–5.2)	0.96	0.46
Vlp9/Vlp12	2.6 (1.2–6.0)	4.0 (1.0–16.6)	0.58	0.88

^a^CI, confidence interval

^b^K-W, Kruskal-Wallis

^c^Bh, *B*. *hermsii*; Bb, *B*. *burgdorferi*

### *In vivo* expression of the *vtp* gene

We used the CC1 strain to infect the mice, because of two-fold higher peak densities in the blood with this strain in comparison to the HS1 strain (unpublished findings). The mice were immunodeficient, which allowed for a proliferation of spirochetes to the carrying capacity without the risk of antibody clearance before blood collection could occur. SCID mice infected for less than a week are not discernibly different in their conditions from immunocompetent mice with infections of this duration [43].

Total RNA in whole blood from a total of 4 infected mice was extracted, treated to remove bacterial ribosomal RNA, and subjected to reverse transcription with random hexamers. Sequencing of the resultant cDNA yielded 484,508 single-end reads of 55 to 255 nt and a mean length of 206 nt. Of these, 315,332 (65%) of the reads mapped to the *Mus musculus* genome. There was ~24x coverage of the mouse mitochondrion genome of 16.3 kb, and ~10x coverage of *B*. *hermsii* strain HS1’s chromosome of 922 kb. Analysis of results for 7 selected mouse genes with expected expression by blood cells showed that the highest ranked mouse genes were beta-globin, alpha-globin, beta actin, and cytochrome oxidase I (S1 Table). For a negative control, there were no measurable transcripts for the pancreatic enzyme trypsin, which would be minimally if at all expressed by blood cells.

**Fig 4:**
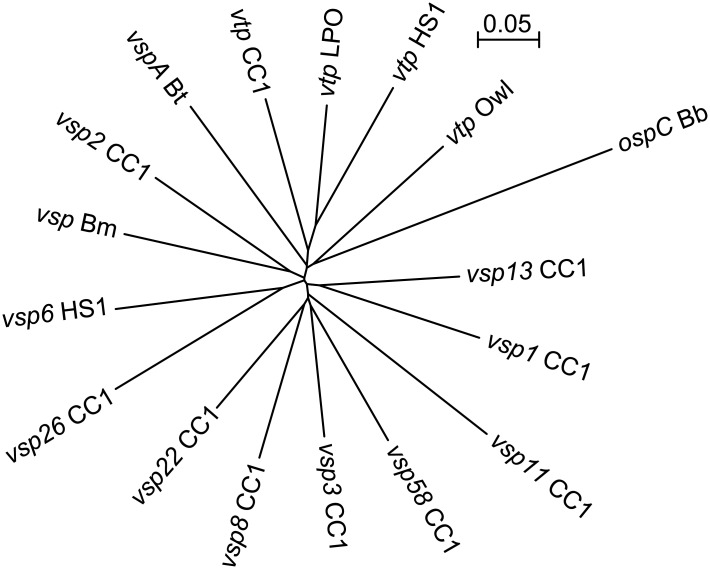
Unrooted neighbor-joining distance phylogram of codon-aligned partial nucleotide sequences of selected *vsp*, *vtp*, and *ospC* genes of strains CC1, HS1, LPO, and Owl of *B*. *hermsii*, strain LB-2001 of *B*. *miyamotoi* (Bm), strain Oz of *B*. *turicatae* (Bt), or strain B31 of *B*. *burgdorferi* (Bb). The accession numbers are given in Table 1, and the sequences for the alignment are given in S1 File. The bar indicates the nucleotide distance.

Table 1 lists the names of sequences and accession numbers of strain CC1, as well as other sequences included in the reference set. In the set were several genes known or suspected to be expressed in vivo by *Borrelia* species: FlaB flagellin [44], the GroEL heat-shock protein [45], the fibronectin-binding protein FbpC [34], GlpQ [46], factor H binding protein FhbA [47], and the intergral membrane protein P66 [48]. Other genes in the set included *recA*, genes for ribosomal proteins (*rplA*, *rplB*, *rplC*, *rpsB*, *rpsC*, and *rpsD*), for cell division proteins (*ftsA* and *ftsY*), and for nucleotide synthesis or salvage (*purA*, *purB*, and *thyX*). The gene sequences for selected VMPs, including the Vtp proteins of two other *B*. *hermsii* strains, LPO and Owl, besides HS1 and CC1, were trimmed of their conserved 5’ ends and 3’ ends for sequences of 443–455 bp (S1 File). Fig 4 is a phylogram showing that the distances between pairs of individual trimmed sequences of these *vtp* and *vsp* genes were near equivalent.

By the criterion of normalized reads per 1000 bp, the most highly expressed genes were *flaB* and *vsp1*, the serotype-defining VMP gene in this population (Table 1). The FlaB flagellin and the expressed VMP are typically the most abundant proteins in whole cell lysates of relapsing fever *Borrelia* species in vitro and in vivo [32, 49, 50], so this finding served to validate the RNA-Seq results. Fourth in rank, after the gene for the heat-shock protein GroEL, was the gene for the Vtp protein of CC1. No reads mapped under these conditions of stringency to the corresponding *vtp* sequences of strains HS1, Owl, or LPO, or to homologous genes of other *Borrelia* species: *vspA* of *B*. *turicatae*, *vsp1* of *B*. *miyamotoi*, and *ospC* of *B*. *burgdorferi*. The reads mapping to some *vsp* genes may be attributable to spontaneous antigenic variation in expanding population, and the subsequent outgrowth of these variant sub-populations in individual infected mice [24]. Further evidence of the specificity of the conditions of the experiment was the lack of reads mapping to strain HS1’s *vsp6* gene, which is absent in strain CC1 [51].

## Discussion

The study’s first part examined whether cells ostensibly free of VMP proteins were in fact producing low amounts of the tick-associated Vtp protein in culture and in vivo. Our supposition about the presence of Vtp in cultivated mutant cells was confirmed by WB analysis with a Vtp-specific monoclonal antibody of a fraction enriched for membrane proteins. We inferred from these findings that full-length Vtp was being expressed either at a low level by the majority of cells or more fully by a minority of cells of a population that expanded from a clone. We did not determine the genetic basis for the unexpected expression of Vtp by the mutant, but here offer two possible explanations. The population injected into the mice began as a cell with a frame shift in a tract of adenines and, as a consequence, a premature stop codon [33]. Occasional errors in transcription may have eliminated in effect the frame shift, thus allowing for full-length translation of some transcripts without genotypic reversion. Alternatively, a second mutation may have restored the wild Vtp^+^ phenotype for a sub-population of cells. Either an insertion of one adenine or a deletion of two adenines at the mutation position would remove the frame shift.

Whatever the genetic basis for residual Vtp expression, the anti-Vtp antibody response to immunization with the mutant was consistent with earlier reports of expression of Vtp by spirochetes in the blood of mice [28, 29]. In these previous experiments the mice were injected with cell populations that prospectively were known to be producing Vtp at time of inoculation. For a different aim, Schwan and Hinnebusch had used the same Vtp-specific monoclonal antibody and indirect immunofluorescence microscopy to document the expression of Vtp (then called “Vmp33”) in the salivary glands of *O*. *hermsi* ticks. But with the same reagent, the authors did not detect Vtp^+^ cells in thin smears of blood from mice infected with wildtype cells of other serotypes in either that or a subsequent study [25, 26]. Could these apparently conflicting sets of results be reconciled? To this end, in the second part of the study we infected mice with small inocula of clonal populations of two different serotypes, 7 and 19, of strain HS1, as well as serotype 1 of another *B*. *hermsii* strain, CC1, which was isolated ~1500 kilometers from the site of the collection the Browne Mountain isolate of HS1.

Infections were initiated by needle injection and not with cultivated cells or by tick bite, the natural route of infection. However, there was justification for using spirochetes in mouse blood, rather than in tick saliva or culture medium, for these particular experiments, which were designed to assess expression of *vtp* in a vertebrate host. If we used as a source either ticks or culture medium—two conditions known to be permissive for Vtp expression [25, 29]—the presence of anti-Vtp antibodies in the sera could be attributable to the initial inoculum and not necessarily to an immune response to bacteria growing in the mouse. By direct transfer of the infection from one mouse to another, the analysis would more specifically be of the mouse environment for the spirochetes, which was the issue of importance.

If we accept at face value the previous reports of absence or very low frequency of Vtp^+^ cells in the blood of mice infected with wildtype VMP^+^ cells [25, 26, 29], how does one account for the observed antibody responses to Vtp in mice infected with other serotypes? It is plausible that the antibodies were elicited by spirochetes preferentially expressing Vtp while in tissues but not in the blood. There is precedence for this conjecture from studies of *B*. *turicatae*, another relapsing fever agent. Two of the serotypes of an otherwise isogenic isolate of *B*. *turicatae* were distinguished not only by their sequence differences but also by their divergent preferences for tissues or blood [52, 53]. Thus, the observed more rapid disappearance of Vtp^+^ cells from blood after infection [26, 28, 29] may be attributable to comparatively greater migration of these cells into tissues. On the other hand, the enumeration of transcripts for a selection of genes (Table 1) provided evidence of the presence of Vtp-expressing cells in the blood of infected mice as well. This was at an overall transcription level of *vtp* about one-tenth of that measured for the serotype-defining *vsp* of the population but still in excess of other *vsp* or *vlp* genes constituting the set.

Cognizant of findings to date, we propose the following model for the contribution of Vtp to *B*. *hermsii* fitness and the role of this protein in structuring this species’ populations. The concept complements Raffel et al.’s model, which specifies the adaptive value of switches between VMP^+^ and Vtp^+^ phenotypes [26] and findings of Vtp-specific immunity against experimental tick-borne infection of mice [54]. Our present understanding is that Vtp is a protein primarily adapted for the tick environment and is critical for transmission from vector to host [25, 26]. But expression of Vtp by spirochetes in the salivary glands means that some cells bearing Vtp proteins in small or moderate amount likely enter the host and there grow to a density sufficient to elicit an antibody response. We note that an inoculum as small as 10^3^ viable but non-infectious *B*. *burgdorferi* elicited antibody responses in mice [55] and that some types of anti-Vtp antibodies were bactericidal even in the absence of complement [26, 56]. A host response of this type conceivably could provide partial or complete immunity for the mammal against infection by that strain in the future, if of little benefit against the ongoing infection. This strain-specific immunity in turn would provide conditions for balancing selection in a shared environment [27, 54], analogously to the circumstances for strains of *B*. *burgdorferi* displaying different OspC proteins [9]. One testable prediction of this model with a systematic field study is existence of sympatry of two or more *B*. *hermsii* strains that are defined in part by antigenically-distinctive Vtp proteins.

## Supporting Information

S1 Fig Unrooted neighor—joining distance phylogram of partial sequences of selected Vtp and Vsp proteins of strains CC1, HS1, LPO, and Owl of *B*. *hermsii*, strain LB-2001 of *B*. *miyamotoi* (Bm), and strain Oz1 of *B*. *turicatae* (Bt). The accession numbers for the coding sequences are given in Table 1, and the coding sequences for proteins in the the alignment are given in S1 Table. The bar indicates amino acid sequence distance.
(EPS)

S1 File Partial sequences of reference set of genes for RNA-Seq and recombinant proteins. (DOC)

S1 Table RNA-Seq of complete coding sequences of selected *Mus musculus* transcripts. (DOC)
