# Correction: Expression of the Tick-Associated Vtp Protein of *Borrelia hermsii* in a Murine Model of Relapsing Fever

**DOI:** 10.1371/journal.pone.0153906

**Published:** 2016-04-13

**Authors:** Renee A. Marcsisin, Eric R. G. Lewis, Alan G. Barbour

There is an error in the current address for the second author, Eric. R. G. Lewis. The correct current address should be: Department of Microbiology and Immunology, University of Texas Medical Branch, Galveston, Texas, United States of America
