# Supplementary material for: Expression of the Tick-Associated Vtp Protein of Borrelia hermsii in a Murine Model of Relapsing Fever
Source: PLoS One. 2016 Feb 26;11(2):e0149889. doi: 10.1371/journal.pone.0149889 (PMC4769344; doi:10.1371/journal.pone.0149889)
Supplement: S1 File — (DOC) [file pone.0149889.s002.doc]

**Supplementary Information File S1**

>Vtp_Bh_HS1

GATTTATCAAAAATAAGTGCAAATATAAAAAACGCTGTTACTTTTGCTGCAAGTGTTCAAGAAGTAGAGA

CCTTAGTTAAGTCTATTGATGAGCTTGCTAAAGCTATTGGACAGAAAGTTAATGCAGATGGTCTTACTGC

TGAAGCAGATAAAAATGATTCATTAGTTGCAGGGGTATATCAATTAATATCAGATGTACAGGGTAAGTTG

ACAAAATTGGAAATTGGGGCCAGCAAGTTTGCTGGACTAAAGGAAAAGGTTGTTGCTGCTAAGAAGGGAA

GTGATGATTTCTTAACCAAAGTAAAAGCGCAGCATAATAATCTTGGTCAAAGTGCTGAAGCCCCAAAGGC

TATAAAGAAAGGTAATGCTGATAGTACTAAGGGAGCTGAAGAACTTGGTAAGTTAAATACAGCAATAGAT

GAGTTGTTAACCGCTGCTAAAGATGCA

>Vtp_Bh_LPO

GATTTATCAAAAATAAGTGCAAATATAAAAAACGCTGTTACTTTTGCTGCAAGTGTTCAAGAAGTAGAGA

CCTTAGTTAAGTCAATAGATGAGCTTGCTAAAGCTATTGGGAAAAAAATTGATGCTAATGGTGGTCTTGC

TGATGAAGCTGATCAAAATGGATCGTTAATTGCAGGAGTACATAGTGTAATATCGGCTGTAAATATTAAA

TTGGGACAATTAGAGAATCAAGAAGGACTTTCTGCTGGCCTTAAGGCCAAAGTTACTGCTGTTAAGAGCG

CAAGTGATAAGTTTAAAGAGAAAGTAAAAGGAGCAAGTGGTGATCTTGGTAAGGCTGCTGCTAAGAGTGA

TGATGCAAAAAAAGCTATAGATCGAAAAAATGGCGCAAAAGATAAAGGAGCTGAAGAGCTTGGTAAGTTG

AATACAGAAATAGATGCGTTGTTAAAGGATGCTAACGATGCA

>Vtp_Bh_CC1

GATTTGTCAAAAATAAGTACAAAAATAAAAGAGGCTGGTGCTTTTGTAGCAAGTGTTAAAGAAGTAGAGA

CCTTAGTTAAGTCAATAGATGAGCTTGCTAAAGCTATTAAAAAGAAAATTCAAGCAGATGGTCTTCAAGA

TGATACTGATAATTTAAATGGAACATTGCTTGCGGGGGCATATCAAATAATGTCTGATGCAGACTCTAAA

TTGACAGCATTGGAGGGTAACTCTGAAAAATTTGCTGGGATGAAGGATAAAGTTACTTCTGCTAAGCAGA

AAAGTACAGCATTTTTAAATAAATTGAAGTCAGAAAATGCTACTCTTGGTGTGGCTTCAGCAGCTGTTTC

TAGTGCTAATGCAAAGGAAGCCATAGATAGAAATAATGCCTCTAAGACTAAAGGGGCAAAAGAGCTTGGT

GAGTTAAATACAGCAATAGATGAGTTGTTAACCGCTGCTAACGATGCA

>Vtp_Bh_Owl

GATTTATCAAAAATAAGTTCAAAGATAAAAAATGCTAGTGCTTTTGCTGCAGGTGTAAAAGAAATTCATG

CCTTAGTTAAGTCAGTAGGTGAATTTGCTAAAGGTATTGGAAATAAAGTGACCCAAAACACTGGTGTTAT

TGATGCTGATGCTGGTGGTAATAATAATGGACAAATAATTGTAGGGGCGTATAGTTTAATATCAGGTTTA

AAGACCCAAGTGGAAGAATTAGGAAAGAAAGATGGAATTTCTGATGGATTGAAGGAAAAGCTTGATGATG

TGAGCAAAAAAGGTAAAGCATTTTTAGATAAAGTGAAGGCAGACGCTGAGCTTTGTAAAAAAGATGTAAC

TGATGAGAATGCAAAAAAAGCCTTGGATGTAAATAATGCTTCTAAAGAGAAAGGGGCTAAAGAACTTGGT

GAACTCGACACAGCAATAGGTGCGTTGTTAAGTGCTGCTAACGATGCA

>Vsp6_Bh_HS1

GATTTGGCAAAGGTAAGTAAAAAGATAAAAGAGGCTAGTGCTTTTGCAGCAAGTGTTAAAGAAGTAGAGA

CTCTAGTTAAGTCAGTAGATGAGCTTGCTAAAGCTATTGGAAAGAAAATTAAAAATGATGATGATGGCTT

TGATACTGAAGCAAATAAAAATGGATCATTACTCGCAGGAACATTGCAATTAATGTTTGCCGTAGGAACT

AAATTGGAATCTTTAGAGAAAATAGCCGGAATTTCTGATGAAGTGAGGGGCAAGGTTATTGTTGTTAAGA

CCGAAAATACAGCATTGATAACTAAATTGAAGGGAGGGGATGCTAGTCTTGGTAAAAATGATGCTTCTGA

TTCTGATGCAAAAAATGCTATAGATAAAAGCGATGTTACTGGTGGCAAAGTAAGGAAGAGCTTATTTAAG

TTAAACACAGCAGTTGATGCCTTGTTAAAGGCAGCTGAGGGTGAA

>Vsp22_Bh_CC1

GATTTGGCAAAGATAAGTAAAAAGATAAAAGAGGCTAGTGCTTTTGCAGCAAGTGTTAAAGAAGTAGAAA

CCTTAGTTAAGTCTATTGATGAGCTTGCTAAAGCTATTGGAAAGAAAATTAAAAATGATGGTATCCTTGA

GTTTGCTGATGCTGATAAAGATAAGAATGGGTCATTAATTGCAGGGGCATTTCAAATAATATTGATTGCG

GAAGGAAAATTGAAAGGCTTAGATAAAGAGGCTGGAATCTCTGAGGCATTAAAGGCAAAGGTTACTGATG

CTGAAGCTAAAAGTAAAGCATTCTTGGCTAAAGTGAAAGGACAAACCGCTACCCTTGGTAAAAATGATGC

TAGTGATGAAGACACAAAAAAAGCTATAGATAGAATAGGACAGCCTGGTGGAGATAAAGGAGCTTCTGAG

CTTGAAGCACTTAACGCAGCAATAGATGAGTTATTAAAGGCTGCTAATGATGCA

>Vsp3_Bh_CC1

GATTTGGCAAAAATAAGTAAAAAGATAAAAGATGCTAGTGATTTTGCAGCAAGTGTTAAAGAAGTTCATA

CTTTAGTTAAGTCTATTGACGAGCTTGCTAAAGCTATTGGAAAGAAAATTAAAAATGATAATAGTAACTT

TGAGGATGAGAATGACCATAATGGATCGTTAATTGCAGGGGTATTTCAAGTAATATTGACTGTAAAAGCT

AAATTAACATCATTAGAGCAAATTATTGGAATTTCTGATGAATTAAAGACAAAGGTTGGCATGGTTAAGA

AAGAAAGTGAAGCATTCGTAACCCAAGTAAAATCAAAGCATACTGATCTTGCTAAAGAAGGTGTTACTGA

TGCACATGCAAAGAGTGCCATACTTGTAACAGATGGCACTAAGGATAAAGGAGCCGCTGAACTTATTAAG

CTCAACACAGCAATAGATGAGTTATTAAAGGCTGCTAATGATGCA

>Vsp2_Bh_CC1

GATTTAGCAAAAATAAGTAAAAAAATAAAAGACGCGGTTGAGTTTGCAGCAAGTGTAAAAGAAATAGAGA

CTTTAGTTAAGTCAATTGATGAGCTTGCTAAAACCATTGGACAGAAGCTTACGAAAGATACAGGTGTTCT

TGCAGCTGATGCTAATAATAATAATGGGGGATTAATTGCAGGGGTATATGGTATAGTAACGGATGTAGGT

ACTAAATTGGACGGGTTATTAAAAGTAAATGGAATTTCTGAAGATATTAAGACAAAAATTAATGATTCTA

AGAGTAAAGGTACAGCATTCTTAAGCAAAGTGAAAGGGGATGATGATCTTTGTAAAAAAGACGCTACAGA

TGCTCATGCAAAAAATGCCATAGATAAAAACGATAATACTGGAGGTAAAGGTAAGACTGAGCTTATTGCC

CTAAACACAGCAATAGATGAGTTATTAAAAGCTGCTAATGAGGCGGT

>Vsp8CC1

GATTTGGCAAAAATAAGTGCAAAGATAAAAGAGGCTAGTACTTTTGTAGCAAGTGTTAAAGAAGTAGAGA

CCTTGGTTAAGTCAATAGATGAGCTTGCTAAAGCTATTGGTAAGAAAATTAAGCAAAATTCCGAAGACCT

TGAAGTGGATAATGGCAAAAATAATAAGAATGGAGAGTTAGTCGCAGGAGCATTTCAGGTAATGTTGACT

GTGAAAGCTAAATTGGAAAAATTAGGGAACACGCCTGAAATTTCTGAAGAGCTAAAAGGAAAGATTACTG

ATTCTAAGAGCAAATGTAAAGAATTTGTGGATAAAGTAAAAGCAGATTCTGATATTTCTAAAGCAGAGGC

TACAGATGAGCATGTAAAAAAAGCTATAGATCAAGTAAATGCTCCTGCTGGAGAAAAAGGTGTTGTTGAA

CTTGTTAAACTTAACAAATCAATAGGTGAGTTGTTAAAGGCTGCTAACGCAGCA

>Vsp58_Bh_CC1

GATTTGGCAAAAATAAGCGCAAAGATAAAAGAGGCTAGTGATTTTGCAACAAGTGTTAAAGAAGTTCATA

CTTTAGTTAAGTCTATTGATGAGCTCGCTAAAGCTATTGGTAAGAAAATCCAAAACGGAGATACTCTTGC

TACTGATAACAATCATAATGGGGGTATAATTGCAGGGTCATTTCAAATGATATCAACTGTAAAGTCTAAA

TTAGACATATTAGCAAAAACAAATGAACTTTCTGATGAATTGAATCAAAAAGTTAATGCTTCTAAAAGCA

AAGCCGACGCTTTCTTAGGTAAATTGAAAACCAATCATACTGATCTTGGTAAAGAAGGAGCTACTGACGA

CCATGCAAAAGCAGCTTTACTTATAACAAATAATACTAAAGATAAAGGAGCTTCTGAGCTTGAAGCACTT

AACGCAGCAATAGATGAGTTATTAAAGGCTGTTAATGCTGCA

>Vsp26_Bh_CC1

GATTTGGCAAAGATAAGTAAAAAGATCAAAGAGGCTAGTGCTTTTGCAGCAAGTGTTAAAGAAATAGAGA

CTTTAGTTAAGTCTATTGATGAGCTTGCTAAAGCTATTGGAAAAAAGATTAAAAATGATGGAACTTTGGA

AGCCATAGCTGATAAGAATGGCTCATTAATTGCAGGGGTGGTTAGTGTTGCTGAAGCTGTGGAAAAAAAA

TTAGGAGAGTTGCAAGTAGCAGGATTCCTTAAAGGCTTAAATGAAAAAGTTCAAGATGTGGATGCTAAGG

TTAAAGCATTTACAAAGAAGTTAAAAGATAAACATGTTGTTTTGGGAGCTGCAGATGGAGCTACTACAGA

TGATAATGCAAAAAAAGCTATAGATCGCGTAAATCAAGTTAATGGGGAGAATGGAGCGGAAGAACTTGGC

AAGCTCAACACAGCAGTTGATGCCTTGTTAAAGGCAGCTGAGGGTGAA

>Vsp11_Bh_CC1

GATTTGGCAAAAATAAGTGCAAAGATAAAAGAGGTTAGTGCTTTTGCAGCAAGTGTAAAAGAAGTTCATA

CTTTAATTAAGTCAATAGGTGATCTTGCCAAAGCTATTGGTAAGAAAATTAAAACAGATGAGACTGGGAC

TTTAGAGTCTTCTACGGCGGATCAAAATGAACAGTTAGTTGCAGGGGCATTTCAAGTGGTATCAACCGTA

AAAGGTGAATTGGAAAGTTTAGTGCAAGTAGATGGAATCTCAGATGACCTTAAGGCGAAGGTTAATGAGG

CCAAGAATGCAAATGATGGTTTATTAAGTAAATTTAAGAGTTCTGCAAAGGATAACGAAAGTGTTAAAAA

AGATGAAGAGGCAAAAAAAGTTATAGATCGAACCAATGCTTCTGCTACTGAGCTTAAGAAACTTGACACA

GCAGTTGATGAGTTGTTAAAAGCTGCTAATGAGGCGGT

>Vsp1_Bh_CC1

GATTTGGCAAAAATAAGTGCAAAGATAAAAGAGGCTAGTGCTTTTGCAGCAAGTGTTAAAGAAGTTCATA

CTTTAGTTAAGTCAGTAGATACGCTTGCTGGTGCTATTGGAAAGAAGATTAAGTCCGATGGGAAGTTTGA

TGCTATGGCTGGTAAGAATGGATCATTGCTTGCAGGGGCATATAATGTTGCGTTGGATATAAATAGTAAA

TTGACAGTATTAGATGGTAAGGCTGGACTCTCTTCTTTACTTAAGGCAAAGGTTACTGCTGCAAAAACTA

GTGGTGAATCATTCTCAAATAAATTAAAAACCGAACATACTGACCTTGGCAAAGAAGAGGCTAGTGATGA

TAATGCAAAAGCAGCTTTACTTGTAACAAATGCTACTAAAAATAAAGGGGTCACTGAGCTTGAAGCACTC

AACACAGCAGTTGATGCCTTGTTAAAGGCAGCTGAGGGTGAA

>Vsp13_Bh_CC1

GATTTGGCAAAAATAAGTAAAAAAATAAAAGATGCTAGTGATTTTGCAACAAGTGTAAAAGAAGTTCATA

CTTTAGTTAAGTCAATAGATGAGCTTGCTAAAGCTATTGGGAAAAAAATTCATAACGATGGTTCTCTTAC

TACTGAAGATGGTAAGAATGGTTCATTACTTGCAGGGGTACATAGTGTAATATCAGCCGTAAAGACTAAA

TTGGGATCATTGGAACAAAAAGCTATTGGAGAATTTGCTGGAATGAAGGTTCAAGTTGTTGCTATTAAGA

CTGCAAGTATAGATTTATTAAATAAATTTAAAGATAAAAATGCTGAACTTGGGAAAAACGAGGTTAGTAA

TGACGATGCGAAAGCTGCCATACTTGTAAGTAATACCACTAAAGATAAAGGAGCTTCTGAGCTTGAAGCA

CTCAACACAGCAATAGATGGGTTGTTAAAGGCTGCTAATGGTGCA

>VspA_Bt_Oz1

GACCTAGCTACAATAACTAAAAACATTACCGATGCTGTTGCTTTTGCTAAGAGTGTTAAAGACGTTCATA

CTTTAGTTAAATCCATTGATGAGCTTGCTAAAGCTATCGGGAAAAAAATTGGTGCCAATGGTCTTGAAAC

TGATGCTGATAAGAATGCAAAATTAATTTCAGGAGCATATAGTGTAATATCAGCTGTAGATACTAAATTA

GCATCATTAGAAAAAAAAGTTGGAATTTCTGATGACTTAAAGGGAAAAATTACTACTGTTAAGAATGCAA

GTACATCCTTTTTAACCAAGGCTAAATCAAAGACAGCTGATCTTGGTAAAGATGATGTTAAGGATGCTGA

TGCGAAGACAGCTATAGATATAGCAGATACTGGAGCCAAGGATAAAGGCGCGGAAGAGCTTATTAAACTC

AATACAGCAATTGATGCTTTGTTAACTTCTGCTGAAGCTGCA

>Vsp1_Bm_LB

GATTTAGTAAAAGTAAGTAAAAAGATAAAAGATGCGGTTGAGTTTGCAGCAAATGTAAAAGAAGTAGAGA

CTTTAGTTAAGTCAATAAATGAACTTGCTAAAGCTATTGGAAAGAAAATTAAGTCCGATGGGCAGTTTGA

TACTGAGTCAGATAAAAATGGATCATTGCTTGCAGGAGCACAAAGTATAATGTTAGCTGTAAAAGCTAAG

TTAGGACAATTGGATAATAAAGAAGGGATTTCTACTGAACTAAAGCAAAAGGTTACTGATTCTAAGACAA

AAACAGAAACTTTCTTAACTAAATTGAAAGACAATCACTCTGATCTTGGTAAAAATGAAGCTACTGATGC

TCATGCAAAAAGTGCTATAGATATAACTGATACTGGTACTAAAGATAAAGGAACTTCTGAGCTTATCGCT

TTGAATACATCAATTAATGCTTTGTTAGAAACCGCTAATGATGAA

>OspC_Bb_B31

AATCTTACAGAAATAAGTAAAAAAATTACGGATTCTAATGCGGTTTTACTTGCTGTGAAAGAGGTTGAAG

CGTTGCTGTCATCTATAGATGAAATTGCTGCTAAAGCTATTGGTAAAAAAATACACCAAAATAATGGTTT

GGATACCGAAAATAATCACAATGGATCATTGTTAGCGGGAGCTTATGCAATATCAACCCTAATAAAACAA

AAATTAGATGGATTGAAAAATGAAGGATTAAAGGAAAAAATTGATGCGGCTAAGAAATGTTCTGAAACAT

TTACTAATAAATTAAAAGAAAAACACACAGATCTTGGTAAAGAAGGTGTTACTGATGCTGATGCAAAAGA

AGCCATTTTAAAAACAAATGGTACTAAAACTAAAGGTGCTGAAGAACTTGGAAAATTATTTGAATCAGTA

GAGGTCTTGTCAAAAGCAGCTAAAGAGATG
