# Supplementary material for: Expression of the Tick-Associated Vtp Protein of Borrelia hermsii in a Murine Model of Relapsing Fever
Source: PLoS One. 2016 Feb 26;11(2):e0149889. doi: 10.1371/journal.pone.0149889 (PMC4769344; doi:10.1371/journal.pone.0149889)
Supplement: S1 Table — (DOC) [file pone.0149889.s003.doc]

S1 Table. RNA-Seq of complete coding sequences of selected *Mus musculus* transcripts

| Rank | Transcript | Length (bp) | Accession no. | Log_10_ reads/1000 bp |
| --- | --- | --- | --- | --- |
| 1 | hemoglobin, beta, adult chain 2 | 394 | XM_011238089.1 | 3.32 |
| 2 | hemoglobin, alpha, adult chain 2 | 429 | NM_001083955.1 | 3.07 |
| 3 | actin, beta | 1128 | NM_007393.3 | 2.80 |
| 4 | cytochrome c oxidase, subunit I | 1545 | AK138996.1 | 2.23 |
| 5 | pyruvate kinase | 1596 | XM_011242674.1 | 1.46 |
| 6 | heme oxygenase I | 870 | NM_010442.2 | 1.40 |
| 7 | trypsin 5 precursor | 741 | NM_001003405.4 | None detected |
